# Investigation of the Gas Permeation Properties Using the Volumetric Analysis Technique for Polyethylene Materials Enriched with Pure Gases under High Pressure: H_2_, He, N_2_, O_2_ and Ar

**DOI:** 10.3390/polym15194019

**Published:** 2023-10-07

**Authors:** Ji-Hun Lee, Ye-Won Kim, Jae-Kap Jung

**Affiliations:** 1 Hydrogen Energy Materials Research Center, Korea Research Institute of Standards and Science, Daejeon 34113, Republic of Korea; ljh93@kriss.re.kr (J.-H.L.); kyw9687@kriss.re.kr (Y.-W.K.); 2 Department of Measurement Science, University of Science and Technology, 217 Gajeong-ro, Yuseong-gu, Deajeon 34113, Republic of Korea

**Keywords:** polyethylene, volumetric analysis technique, gas permeability, amorphous phase, fractional free volume

## Abstract

Polyethylene (PE) is widely used as a gas-sealing material in packing films and gas transport pipes. A technique for evaluating the permeability of water-insoluble gases has recently been developed. This technique is a volumetric analysis that is used to calculate the gas permeability by measuring the gas uptake and diffusivity. With this technique, we investigated the permeability of pure gases, such as H_2_, He, N_2_, O_2_ and Ar, enriched under high pressure up to 9 MPa in low-density polyethylene (LDPE), ultrahigh molecular weight polyethylene (UHMWPE) and high-density polyethylene (HDPE). The gas uptake showed a linear pressure-dependent behavior that followed Henry’s law, and the diffusivity was independent of the pressure. Furthermore, the logarithmic diffusivity values of the five gases linearly decreased as their molecular kinetic diameters increased. The logarithmic solubility values linearly increased as the critical temperatures of the gases increased. The calculated permeability results were correlated with the volume fraction of the amorphous phase and the fractional free volume. This result newly showed that the amorphous phase was directly correlated to the fractional free volume.

## 1. Introduction

Polyethylene (PE) materials play a crucial role in a wide range of industrial applications across a variety of sectors, including packaging, electronics, gas transportation, storage and medical devices [1,2,3,4,5,6,7]. In the packaging industry, PE ensures product safety, extends shelf life and contributes to a sustainable supply chain due to its versatility, cost-effectiveness and excellent barrier properties [8]. In the electronics industry, PE materials contribute to safe and efficient power transmission due to their outstanding insulating properties [9]. The benefits of using PE in the area of gas transportation and storage include leakage sealing, durability, flexibility, lightweight properties, high chemical resistance and environmental sustainability [10,11]. These advantages make PEs appropriate candidates for gas transportation and storage.

In the fields of gas transportation and storage, studies on the gas-barrier properties of PE have been conducted over a long period of time to prevent the leakage and waste of gases [12,13,14]. Recently, medium-density polyethylene (MDPE) and high-density polyethylene (HDPE) pipelines for the transport of pressurized methane–hydrogen mixture gas have gained interest as highly cost-effective candidate materials for lower permeation [15]. In addition, HDPE is used as a liner of Type IV fuel storage tanks in 70 MPa high-pressure hydrogen environments for lightweight fuel cell electric vehicles [16,17]. In a high-pressure hydrogen environment, rapid depressurization destroys the PEs and removes the sealing ability. This phenomenon occurs because the high-pressure gas accumulates inside the PE due to the permeability of gas. Therefore, to evaluate the effect of high-pressure gas on PEs, the gas permeation phenomenon needs to be understood.

Meanwhile, gas permeation of polymers is the process by which gases diffuse through polymer materials. Gas permeation properties are critical to the applications such as gas separation and packing materials, because they determine the ability of a polymer to selectively allow the permeation of particular gases or prevent gas permeation, respectively.

The permeation of gas molecules into polymers is primarily a function of the polymer structure, gas penetrant, temperature and applied pressure gradient [7]. In particular, the relationship between the polymer structure and gas permeability has been extensively investigated. According to the related literature [18,19,20,21,22], gas permeability is strongly associated with chain mobility and chain packing of polymers. Mousavi et al. [23] stated that the gas permeability of PE was not affected by the sample thickness and that the permeability decreased with increasing crystalline phase fraction. Fujiwara et al. [16] examined the permeability of pressurized hydrogen up to 90 MPa in PEs of various densities and reported a relationship among permeability, crystallinity and free volume fraction. The relationship between the fraction of the amorphous phase and the permeation properties of various gases for PE materials was observed by Flaconnèche et al. [24]. Additionally, researchers discovered that the solubility was related to the critical temperatures of the gases and that the diffusivity was inversely proportional to the kinetic diameters of the gas molecules [24,25]. The kinetic diameter is the size of the molecules based on the possibility of collisions and can be determined by using an intermolecular model, such as the Lennard–Jones diameter [26].

However, there are few studies on the permeation properties of various high-pressure gases undertaken in a systematic manner, including permeation research of hydrogen gas under high pressure. The understanding of the permeation properties of pressurized gas, including hydrogen with the lowest molecular weight, is important. From this point of view, we investigated the effect of five different readily available gases (H_2_, He, N_2_, O_2_ and Ar) on the permeation properties (diffusivity, solubility and permeability) of PE materials up to a pressure of 9 MPa. These gases were also selected owing to an appropriate wide range in the kinetic diameters and critical temperature, to investigate the effect of gas type on diffusivity and solubility.

In this work, a volumetric analysis technique using a graduated cylinder that was recently developed by our group was used to measure the volume of gas released from gas-enriched polymeric materials. This technique could compensate for the minute variations in temperature/atmospheric pressure to determine the amount of released gas and the gas permeation properties of PE materials using a diffusion analysis program [27]. In our study, we provided a database on the gas permeation properties of the PE materials with different densities for the five types of pure gases. Low-density polyethylene (LDPE), ultrahigh molecular weight polyethylene (UHMWPE) and HDPE were selected as the experimental PE materials, whereas pure H_2_, He, N_2_, O_2_ and Ar, at a pressure range of 3–9 MPa, were chosen as the five testing gases. The dependence of the pressure and sample thickness on the permeation properties were investigated for the five testing gases. The diffusivity and solubility of PE were related to the kinetic diameter and critical temperature of the gas molecules, respectively. Clear correlations between the permeation properties and volume fraction of the amorphous phase were observed for the PE materials. From the investigation on the effects of the volume fraction of the amorphous phase, we quantitatively determined the permeation properties of the 100% amorphous phase from PEs with different densities. The permeability in the PE materials exponentially increased with increasing fractional free volume. Finally, a novel concept was provided to derive a new correlation between the amorphous phase and free volume from the permeability data.

## 2. Materials and Methods

### 2.1. Sample Preparation and Gas Exposure Conditions

Experiments were conducted by using three commercially available PE varieties, i.e., LDPE, UHMWPE and HDPE, with 300 × 300 mm square sheets. LDPE and HDPE were produced by extrusion molding, and UHMWPE was produced by skiving [28]. To evaluate the gas permeation properties with respect to the thicknesses of the samples, the samples were prepared as follows:

- Cylindrical-shaped LDPE, UHMWPE and HDPE with radii of 9.5 mm and thicknesses of 1.64, 3.24 and 4.93 mm, respectively.

**Figure 1:**
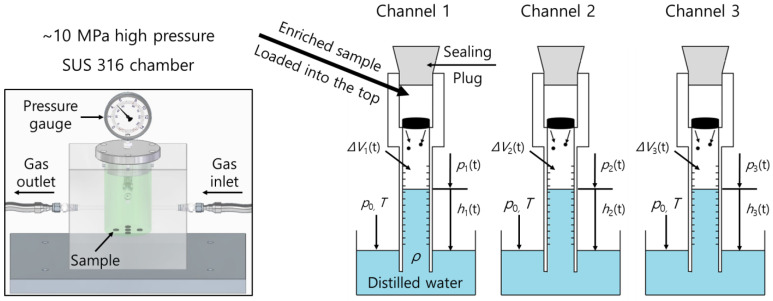
Schematic representation of the three-channel volumetric analysis system after gas charging to 10 MPa and subsequent decompression. The blue color indicates distilled water.

The gases were charged using a stainless steel 316 chamber with an inner diameter of 50 mm and height of 50 mm. Samples were exposed to 25 °C at a fixed pressure ranging from 3 MPa to 9 MPa. The samples were placed inside the chamber, and the chamber was sealed with the valves closed and purged three times with the corresponding gas at 1–2 MPa. The chamber was pressurized to the experimental pressure and charged until equilibrium was reached for gas absorption. H_2_ and He gases were charged for 24 h due to their fast diffusion rates, whereas N_2_, O_2_ and Ar gases were charged for 48 h due to their slow diffusion rates [27]. After charging, the valve was opened, and the pressure inside the chamber was reduced to atmospheric pressure. The instant at which the chamber pressure reached atmospheric pressure was t = 0, and the time elapsed from this point was recorded. Since the sample was removed from the chamber in 2–5 min and then loaded into the graduated cylinder in Figure 1, time delay (lag) occurred until the measurement began.

### 2.2. Measurement of Density and Crystallinity

The densities of the PE materials were determined by separately measuring the mass and volume of the material. The mass was determined using an electronic balance with a resolution of 0.01 mg, while the sample diameter and thickness were determined using a Vernier caliper with a resolution of 0.01 mm for sample volume calculation.

Differential scanning calorimetry (DSC; SETARAM DSC 131 EVO, Caluire, France) was utilized to determine the degree of crystallinity. From the PE sheet, a sample for DSC testing with a mass of 10.0 ± 0.5 mg was removed. Three reference materials of indium, tin and zinc were used for temperature and caloric calibration. In an Ar environment, the temperature was increased in 10 °C intervals each minute from room temperature to 200 °C. To eliminate the influence of processing conditions, the PE material was recrystallized and then evaluated for crystallinity by a second heat scan.

The degree of crystallinity in PE materials was the ratio of the latent heat of fusion of the PE sample to the latent heat of fusion of 100% crystalline polymer and was calculated as follows [29,30]:

(1) $$Crystallinity\left(\%\right)=\frac{\Delta Heat}{293}$$

where the latent heat of fusion of the 100% crystalline polymer was 293 J/g, and Δ*Heat* (J/g) represents the latent heat of fusion of the PE sample used for measurement. The density and degree of crystallinity determined through this method are listed in Table 1 with their measured uncertainties.

### 2.3. Measurement of Emitted Gas Concentration

We recently developed a volumetric analysis technique for measuring the volume and concentration of gas emitted from polymeric materials with enriched gas (Figure 1). The gas absorbed inside the polymer under high pressure conditions was released to the outside due to the pressure difference formed when the surrounding environment was reduced to atmospheric pressure.

As depicted in Figure 1, the volumetric method was based on a measurement of the emitted gas volume change through three channels using three graduated cylinders. After depressurization, the sample containing enriched gas was loaded into the empty space at the top of each channel and then sealed with a silicon plug. The emitted gas then pushed the water toward the bottom, and a decrease in the water level was detected. The internal pressure, emitted gas volume and water level of each channel were changed and recorded in real time.

The internal pressure of the graduated cylinder for the *i*-th channel, *p_i_*(*t*), was expressed by the principles for a U-tube liquid manometer, as follows [31]:

(2) $$p_{i}\left(t\right)=p_{0}-\rho gh_{i}\left(t\right),i=1,2,3$$

where *p*_0_ is the atmospheric pressure outside the cylinder, *ρ* is the density of water at 25 °C and 997 kg/m^3^, g is the acceleration of gravity, 9.80 m^2^/s, and *h_i_*(*t*) is the water level for the *i*-th channel.

The number of moles (*n*) of gas inside the graduated cylinder followed the ideal gas equation, *pV* = *nRT*, where *R* is the ideal gas constant of 8.314 × 10^−6^ m^3^∙MPa/(mol∙K) and *T* is the absolute temperature. Eliminating the volume occupied by the sample and the water level from the total internal volume yielded the volume of the emitted gas. Since there was already a volume occupied by air before the sample was outgassed in the sealed graduated cylinder, the volumetric change was measured to determine the moles of gas added to each cylinder.

(3) $$n_{i}\left(t\right)=\frac{p_{i}\left(t\right)V_{i}(t)}{RT},i=1,2,3$$

where $n_{i}\left(t\right)$ is the molar change inside the *i*-th cylinder and $V_{i}(t)$ is the volume change by emitted gas inside the *i*-th cylinder.

The amount of gas increased in each cylinder was expressed as the mass concentration [*C_i_*(*t*)] of gas released from the sample, as follows:

(4) $$C_{i}\left(t\right)\left[wt\cdot ppm\right]=n_{i}(t)\left[mol\right]\times \frac{M_{gas}\left[\frac{g}{mol}\right]}{m_{s}\left[g\right]}\times 10^{6},i=1,2,3$$

where *M_gas_* [g/mol] is the molar mass of the gas molecules and *m_s_* [g] is the mass of the sample. Thus, $V_{i}(t)$, which corresponds to the water level change *h_i_*(*t*) with respect to time elapsed after depressurization, was measured by Equations (3) and (4) to obtain the mass concentration of the released gas.

### 2.4. Diffusion Analysis Program for Determining Gas Uptake and Diffusivity

When gas molecules dissolved into a polymer under high pressure conditions are depressurized to atmospheric pressure conditions, these gas molecules from the polymer are initially rapidly and then slowly emitted over time. The gas molecules obey Fickian diffusion laws, and the gas mass concentration *C*(*t*) from a cylindrical sample is expressed by Equation (5) under the boundary conditions, with an initially constant gas concentration at the cylindrical surface of the sample [32,33].

(5) $$C\left(t\right)=C_{\infty }\left(1-\frac{32}{\pi^{2}}\times \left[\sum_{n=0}^{\infty }\frac{exp\{-\frac{{\left(2n+1\right)}^{2}\pi^{2}Dt}{l^{2}}\}}{{\left(2n+1\right)}^{2}}\right]\times \left[\sum_{n=1}^{\infty }\frac{exp\{-\frac{D{\beta_{n}}^{2}t}{r^{2}}\}}{{\beta_{0,n}}^{2}}\right]\right)$$

For a cylindrical coordinate system with axial symmetry, Equation (5) provides the solution to Fick’s second law of diffusion. The total quantity (mass concentration) of gas emitted over an infinite period is denoted by *C*_∞_ (gas uptake), while the diffusivity is given by *D*. *β*_0,*n*_ is the root of the zero-order Bessel function [34], l is the thickness of the cylindrical sample and *r* is the radius of the sample.

To evaluate the mass concentration data of the emitted gas as a function of time using Equation (5), we developed a diffusion analysis program that could compute to the 50th term [35]. This ability enabled the automatic and precise calculation of *C*_∞_ and *D* and accounted for the quantity of gas discharged during the delayed time.

**Figure 2:**
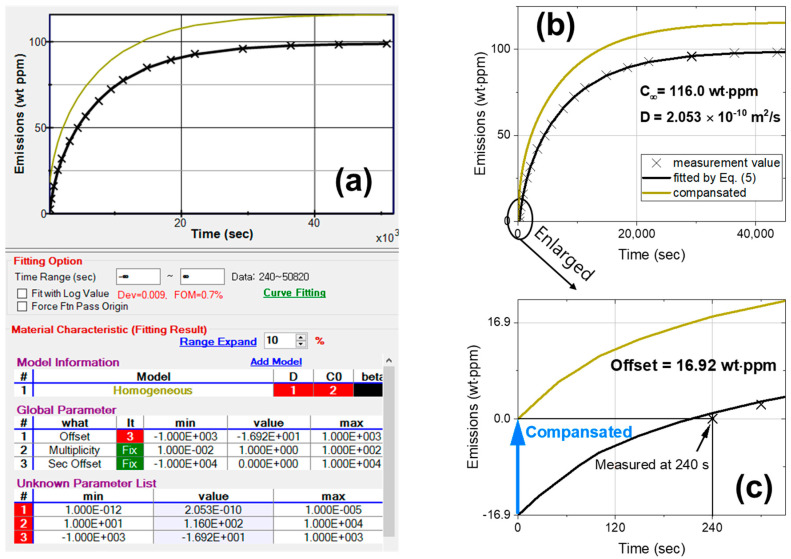
(**a**) Diffusion analysis program for evaluating the emission content and diffusivity of hydrogen using Equation (5); (**b**) measurement data (marked with ×) fitted by Equation (5) (black line) and the offset-compensated result (yellow line); (**c**) enlarged graph of the compensation.

After measuring gas emissions from the polymer samples, a diffusion analysis program was utilized to evaluate *C*_∞_ and *D*. Figure 2 shows an analysis example of measured data where measurements start at 240 s after decompression. Figure 2a shows the frame of the diffusion analysis program, where the radius and thickness of the cylindrical sample in emission mode are used to fit the data (marked with ×) to the black line curve. The diffusion analysis program showed a total of three results; (1) *D* (diffusivity), (2) C0 (gas uptake, *C*_∞_) and (3) offset. The evaluated values were as follows: *D* = 2.053 × 10^−10^ m^2^/s, *C*_∞_ = 116.0 wt∙ppm and offset = 16.92 wt∙ppm, as shown at the bottom of Figure 2a after the optimization of the parameters using the least squares method. The figure of merit (FOM) value (0.7% in Figure 2a) represents the fit result between the data and Equation (5), with a smaller FOM indicating a better fit.

In an actual situation, gas was immediately emitted after depressurization; thus, the concept of offset was introduced to compensate for the area between the black fitting curve and the yellow curve passing the origin in Figure 2b. If the measurement was initiated after 240 s of depressurization, the measured quantity of gas emitted at 240 s was zero. As shown in the enlarged Figure 2c, the offset value (16.9 wt∙ppm) indicated the amount of gas loss due to the time delay of 240 s. Thus, an offset of 16.9 wt∙ppm was used to compensate for a *C*_∞_ of 116.0 wt∙ppm.

## 3. Results and Discussion

### 3.1. Effect of Pressure and Gases on Gas Uptake/Diffusivity

**Figure 3:**
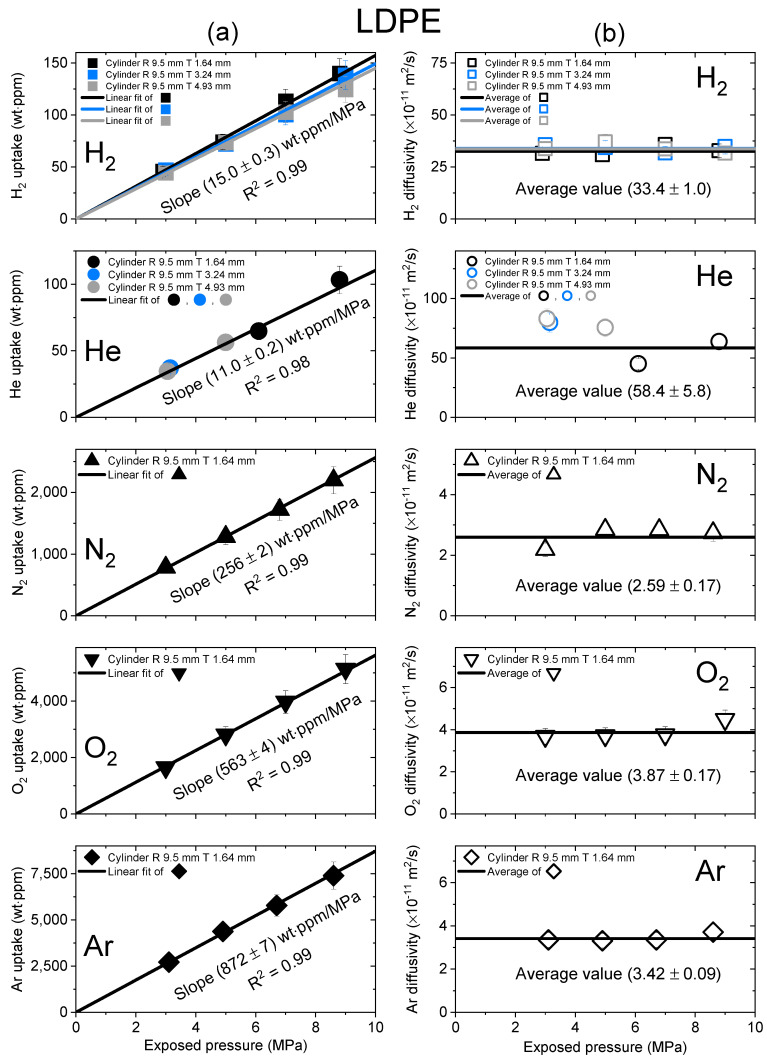
(**a**) Gas uptake; (**b**) diffusivity as a function of exposed pressure for the five gases in a cylindrical-shaped LDPE with different thicknesses. R and T represent the radius and thickness of cylindrical-shaped LDPE, respectively.

**Figure 4:**
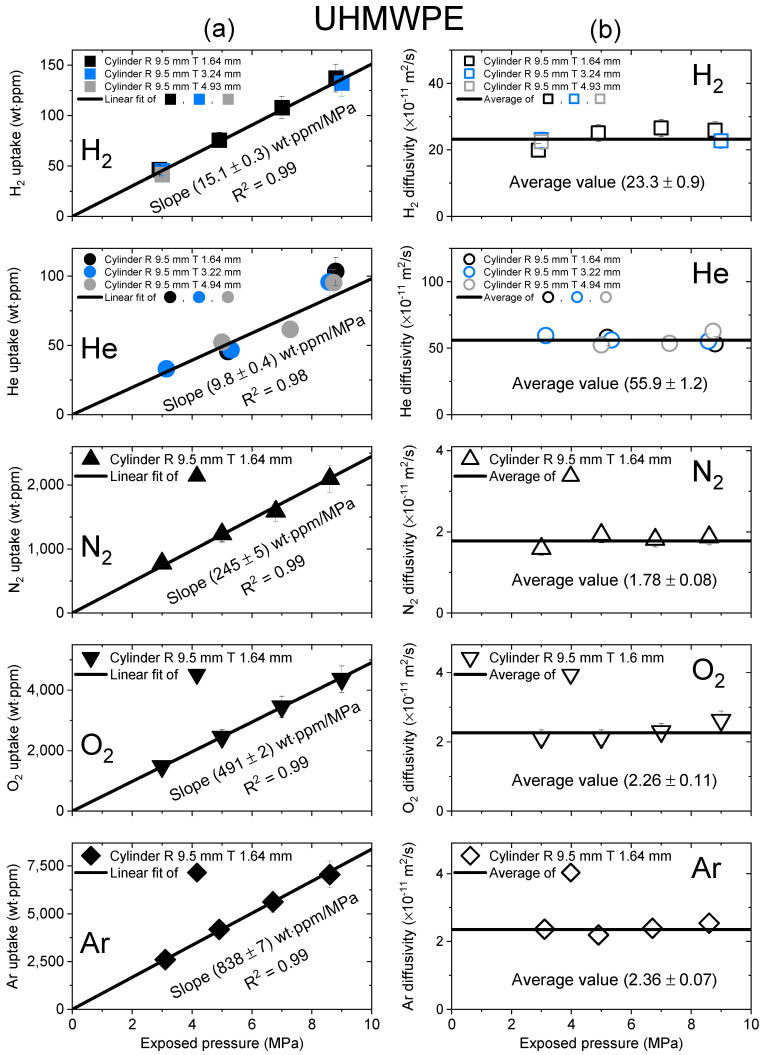
(**a**) Gas uptake; (**b**) diffusivity as a function of exposed pressure for the five gases in a cylindrical-shaped UHMWPE with different thicknesses. R and T represent the radius and thickness of the cylindrical-shaped UHMWPE, respectively.

**Figure 5:**
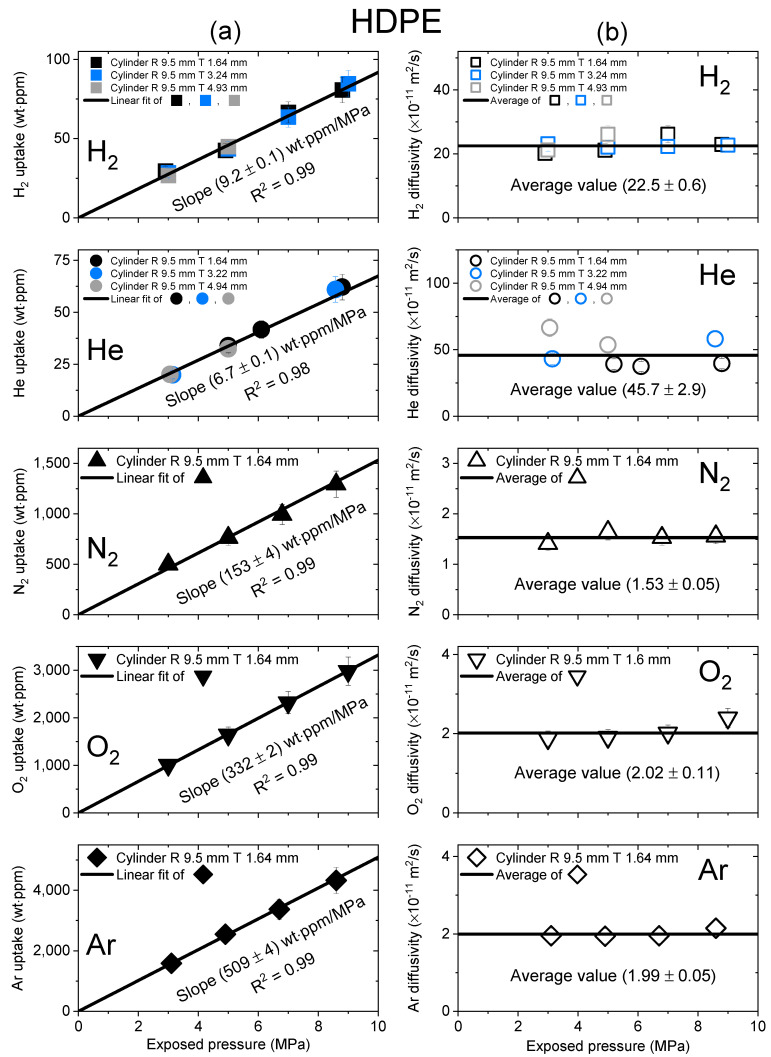
(**a**) Gas uptake; (**b**) diffusivity as a function of exposed pressure for the five gases in a cylindrical-shaped HDPE with different thicknesses. R and T represent the radius and thickness of the cylindrical HDPE, respectively.

Figure 3, Figure 4 and Figure 5 show the gas uptake/diffusivity versus the exposed pressure in LDPE, UHMWPE and HDPE, respectively, for the five gases. The gas uptakes and diffusivities were determined by applying Equation (5) to a diffusion analysis program in Figure 2. All gas uptakes followed Henry’s law up to pressures of 9 MPa, which was consistent with previous investigations [19,27,36]. The slope of the gas uptake data line regarding exposed pressure provided the Henry’s law constant.

Figure 3 shows the gas uptake and diffusivity of LDPE as a function of exposed pressure for five distinct gases. As shown in Figure 3a, the black, blue and gray lines fitted to the gas uptake data had square correlation coefficients of R^2^ > 0.99, indicating that LDPE absorbed the gas molecules in their molecular state without undergoing dissociation or chemical reactions. As shown in Figure 3b, the diffusivity did not exhibit a substantial dependence on the exposed pressure. Thus, the average diffusivity was used, as indicated by the black horizontal line. The black error bars in uptake and diffusivity represented the expanded measurement uncertainty of 10%, as estimated in an earlier study [27]. The two hydrogen results in Figure 3a,b show comparisons of the uptake and diffusivity values for LDPE samples with varying thicknesses. The hydrogen gas uptake and diffusivity values coincided within measurement uncertainty, regardless of sample thickness. Thus, the slope of hydrogen gas uptake for the exposed pressure was equal to the average slope value (15.0 ± 0.3 wt∙ppm/MPa), and the diffusivity was equal to the average value of the data points with different thicknesses.

Figure 4 shows the gas uptake and diffusivity versus the exposed pressure for UHMWPE for the five gases. The gas absorption behavior of UHMWPE in Figure 4a followed Henry’s law to a maximum of 9 MPa, as indicated by the black line, with an R^2^ value greater than 0.98. As shown in Figure 4b, the diffusivity was not pressure dependent. Hence, the average diffusivity was used, as indicated by the black horizontal line.

Figure 5 shows the gas uptake and diffusivity of HDPE versus exposed pressure for five gases. The black line representing HDPE in Figure 5a had a correlation coefficient R^2^ value of 0.99; this result indicated that the relationship effectively followed Henry’s law to a maximum value of 9 MPa. As shown in Figure 5b, the diffusivity did not depend on pressure; therefore, an average diffusivity was also used and indicated by the black horizontal line.

For accurate measurements for all gases, thick samples were needed for the H_2_/He gases due to their fast diffusion characteristics, and only thin samples were needed for the N_2_/O_2_/Ar gases due to their slow diffusion rates. According to Equation (5), if *C*_∞_ and *D* are constant for the same gas and sample, the square of the sample thickness is proportional to the time. Thus, we investigated the linear relationship between the square of the sample thickness and the equilibrium time to verify that the sample thickness did not truly affect the experimental results; specifically, the equilibrium time occurred when the gas concentration, *C*(*t*), reached 97% of *C*_∞_ [*C*(*t*)*/C*_∞_ = 0.97].

**Figure 6:**
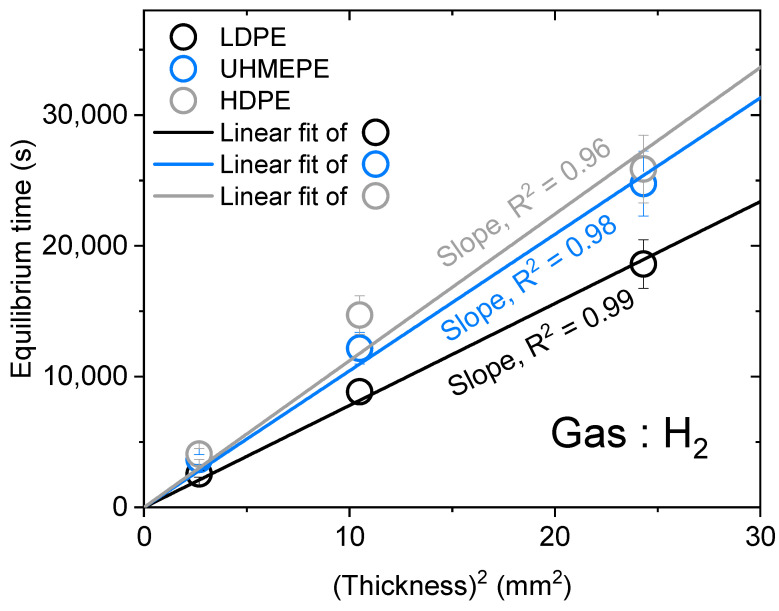
Linear relationship between the squared thickness of the specimen and the equilibrium time.

In Figure 6, the linear correlation passing the origin of the equilibrium time vs. the square of the sample thickness had an R^2^ value greater than 0.96, indicating a correlation between these two parameters. Thus, even if the sample thickness was different, the measurement results (gas uptake/diffusivity) were constant.

Moreover, the solubility (*S*) could be computed from the slope value in Figure 3a, Figure 4a and Figure 5a, as follows:

(6) $$S\left[\frac{mol}{m^{3}\cdot MPa}\right]=\frac{Slope\left[\frac{wt\cdot ppm}{MPa}\right]\times d_{PE}[\frac{g}{{cm}^{3}}]}{M_{gas}[\frac{g}{mol}]}$$

where *d_PE_* is the density of the sample and *M_gas_* is the molecular weight of the test gas. Thus, the measured diffusivity and computed solubility values of LDPE, UHMWPE and HDPE for the five gases are summarized in Table 2. The estimated relative expanded uncertainty value amounted to 10% for both the diffusivity and solubility measurements.

**Figure 7:**
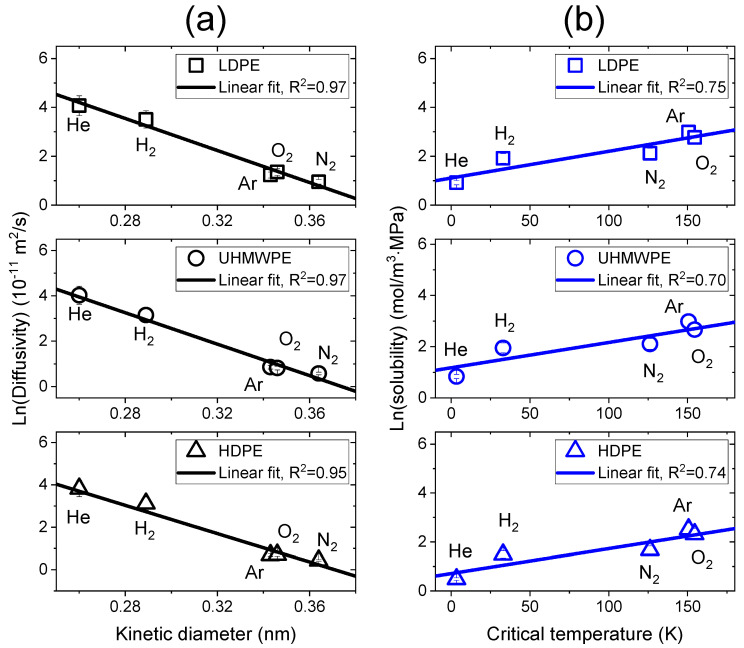
(**a**) Logarithmic diffusivity vs. the kinetic diameter of the gas molecules; (**b**) logarithmic solubility vs. the critical temperature of the gas molecules for the five gases in LDPE, UHMWPE and HDPE. The black and blue lines indicate linear correlations between parameters.

The diffusivity and solubility could be correlated with the kinetic diameter of gas molecules and with the critical temperature of the gas, respectively [25,37,38,39,40]. The kinetic diameter and critical temperature values of gas molecules are represented in Table 3 [24,26,27]. Figure 7 shows the correlation between the diffusivity and the kinetic diameter and between the solubility and the critical temperature in LDPE, UHMWPE and HDPE for the five gases. This result was in good agreement with the trend from previously reported results [24,27,41]. As shown in Figure 7a, the logarithmic diffusivity values of all samples linearly decreased with increasing kinetic diameters of the gases (R^2^ > 0.95). The solubility in Figure 7b was linearly related to the critical temperatures of the gas molecules. Generally, the solubility is affected by many factors, such as gas condensability, polymer crystallinity and polymer–gas molecular interactions [39,40]. Although there are several factors related to the solubility, the logarithmic solubilities of all samples linearly increased with increasing critical temperatures of the gases.

### 3.2. Effects of the Amorphous Phase and Free Volume on the Gas Permeation Properties

Many studies describe the gas permeation mechanism as a solution–diffusion mechanism [42,43,44]. This process assumes that the gas permeability is influenced by two independent factors: the solubility (*S*) and the diffusivity (*D*). Thus, permeability (*P*) is calculated as follows:

(7) P=SD

Moreover, permeability in polymers is related to the internal crystalline and amorphous phases. By assuming that PE exists in crystalline and amorphous phases, the volume fraction of the amorphous phase is known as an important factor affecting permeability [5,16,17]. The volume fraction of the amorphous phase (*φ_a_*) can be calculated from the density of PE and the crystallinity measured by DSC in Table 1, as follows:

(8) $$\varphi_{a}(\%)=\frac{1-Crystallinity}{100}\times \frac{d_{PE}}{d_{a}}$$

where *d_PE_* is the density of PE materials [g/cm^3^] and *d_a_* is the density of 100% amorphous PE, which is 0.855 g/cm^3^ [45].

Meanwhile, the gas molecules between the amorphous phases connecting infinitely are only diffused without blockages in the PE network. In view of the microstructure, the crystalline phase is randomly distributed in the PE network and the penetrating gases are insoluble/non-diffusible in the crystalline phase [13,14,16,46,47,48,49]. Thus, at a 100% crystalline phase $\left(\varphi_{a}=0\right)$, the gas permeability becomes zero. Lasoski and Cobbs [50] assumed that solubility and diffusivity were linear functions of *φ_a_*, as follows:

(9) $$S=S_{a,j}\varphi_{a}$$

and

(10) $$D=D_{a,j}\varphi_{a}$$

Thus, the permeability in Equation (7) can be obtained by multiplying the diffusivity and solubility.

(11) $$P=SD=S_{a,j}D_{a,j}\varphi_{a}^{2}=P_{a,j}\varphi_{a}^{2}$$

where $S_{a,j},\;D_{a,j}\;and\;P_{a,j}$ are the solubility, diffusivity and permeability, respectively, of 100% amorphous PE $\left(\varphi_{a}=1\right)$, with *j* signifying the test gases.

**Figure 8:**
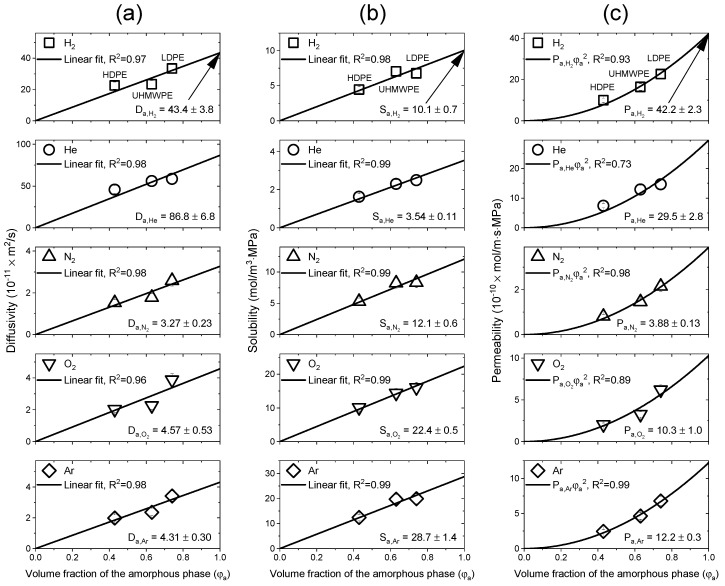
Process of calculating the diffusivity, solubility and permeability values of the 100% amorphous PE for the five test gases: (**a**) Linear fit of the diffusivity for the volume fraction of the amorphous phase via Equation (10); (**b**) linear fit of the solubility for the volume fraction of the amorphous phase via Equation (9); (**c**) quadratic fit of the permeability for the volume fraction of the amorphous phase via Equation (11).

Figure 8a–c shows the diffusivity, solubility and permeability, respectively, of the three samples for the five gases as functions of the volume fraction of the amorphous phase under the assumption that the diffusivity and solubility are zero when $\varphi_{a}=0$. The obtained results were consistent with Equations (9)–(11). Figure 8a shows the process for obtaining the diffusivity of the 100% amorphous phase PE ($D_{a,j})$ by extrapolating a straight line passing through the origin using Equation (10). In Figure 8b, the solubility of 100% amorphous PE ($S_{a,j})$ was obtained by the same process using Equation (9). In Figure 8c, the permeability was interpreted as a quadratic function passing through the origin according to Equation (11), and the $P_{a,j}$ value was also obtained.

The magnitude of $P_{a,j}$ in PE decreased in the order of $P_{a,H2}$ > $P_{a,He}$ > $P_{a,Ar}$ > $P_{a,O2}$ > $P_{a,N2}$. Based on the effect of gas size, for the small gas molecules (H_2_ and He), diffusivities and solubilities dominated the effect on permeability; however, the permeabilities for the relatively larger gas molecules (N_2_, O_2_, and Ar) were dominated by the solubility [24,46,51].

Moreover, many scholars have reported that gas permeation in polymers occurs in the free volume formed by polymer chain mobility [25,51,52,53]. The fractional free volume (FFV) can be determined using the following equation:

(12) $$FFV=1-\frac{d_{PE}}{d_{o}}$$

where *d_PE_* [g/cm^3^] is the density of PE and $d_{o}=1.366\;g/{cm}^{3}$ and is the occupied density of PE obtained by the group contribution method [17,25,40]. The permeability is expressed as an exponential function of FFV, as follows:

(13) $$P=P_{FFV,j}\cdot exp\left(\frac{-B_{j}}{FFV}\right)$$

where $P_{FFV,j}$ is related to the sizes and shapes of the gas molecules, $B_{j}$ is determined by the type of gas and the free volume size in the polymer, $P_{FFV,j}$ has the same unit as permeability [mol/(m∙s∙MPa)] and $B_{j}$ is a zero-order constant.

**Figure 9:**
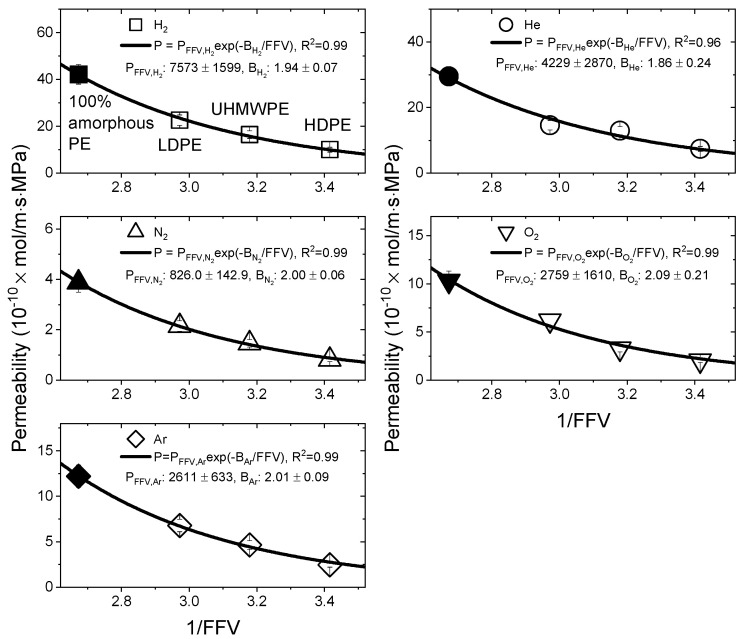
Correlation between the permeability and reciprocal fractional free volume values for PE materials. The $P_{FFV,j}$ and $B_{j}$ values computed by Equation (13) for the test gases are shown in the graph. The black-filled symbols indicate the $P_{a,j}$ values calculated by Equation (11).

According to Equation (13), the correlation between 1/FFV and permeability is shown in Figure 9. The unfilled symbols represent the experimental data points. The 1/FFV value of 100% amorphous PE was 2.673, and the black-filled symbols indicate the $P_{a,j}$ values calculated by Equation (11) in Figure 8. The magnitude of $P_{FFV,j}$ decreased in the order of $P_{FFV,H2}$ > $P_{FFV,He}$ > $P_{FFV,Ar}\;\approx \;P_{FFV,O2}$ > $P_{FFV,N2}$, and this result was similar to that of $P_{a,j}$. Regardless of the test gases, $B_{j}$ appeared to be constant, with an average value of 1.98 ± 0.13.

The permeation parameters $D_{a,j}$, $S_{a,j}$, $P_{a,j}$, $P_{FFV,j}$ and $B_{j}$ obtained in Figure 8 and Figure 9 are summarized in Table 4.

From the results in Figure 8c and Figure 9, the permeability decreased as the volume fraction of the amorphous phase and FFV decreased. This finding showed that the reduction in the amorphous phase was closely related to the reduction in the free volume. A recent study reported that the free volume decreased when the amorphous phase of PE was compressed by the hydrostatic pressure of the gas [17]. Thus, the following equation was used to investigate the correlation and obtained through Equations (11) and (13).

(14) $$P=P_{a,j}\varphi_{a}^{2}=P_{FFV,j}\cdot exp\left(\frac{-B_{j}}{FFV}\right)$$

where $P_{a,j}$ and $P_{FFV,j}$ have the same units as the permeability, and $\varphi_{a}^{2}$ and $exp\left(-B_{j}/FFV\right)$ are dimensionless constants.

**Figure 10:**
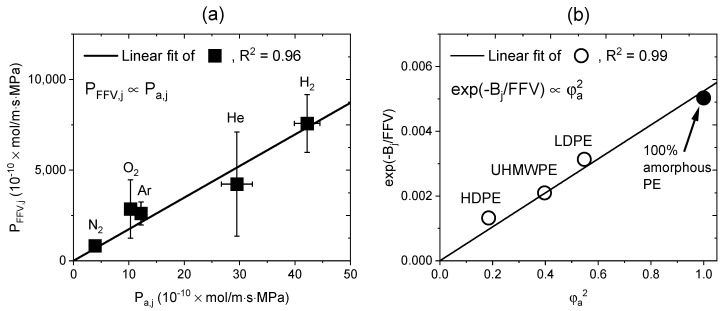
Correlation between the same-dimensional amorphous phase volume parameters and free volume parameters: (**a**) $P_{a,j}$ versus $P_{FFV,j}$; (**b**) $\varphi_{a}^{2}$ versus $exp\left(-B_{j}/FFV\right)$.

Thus, the relationship between the parameters in Equation (14) are shown in Figure 10. From Figure 10a, a linear correlation between $P_{a,j}$ and $P_{FFV,j}$ was obtained for the five gases. $P_{FFV,j}$ was expressed as a function of the permeating gas type [53] and had a linear correlation with $P_{a,j}$. The value of $B_{j}$ was the same as the average value of 1.98 ± 0.13 for the five gases, and the correlation between $\varphi_{a}^{2}$ and $exp\left(-B_{j}/FFV\right)$ is shown in Figure 10b, with an R^2^ value of 0.99, indicating a good correlation. Thus, the observed permeability decreases for the PE specimens with different densities; this was closely related to the reduction in the free volume caused by the reduced volume fraction of the amorphous phase.

## 4. Conclusions

The permeabilities of H_2_, He, N_2_, O_2_ and Ar gases through three PE materials with different densities were investigated by using a volumetric analysis technique using a graduated cylinder and a precise diffusion analysis program. This technique was used to simultaneously determine the permeability, diffusivity and solubility by quantifying the concentration of gas released from gas-enriched polymers after high-pressure charging and subsequent depressurization.

The experiment and analysis showed that the gas uptake for LDPE, UHMWPE and HDPE followed Henry’s law up to 9 MPa, supporting the pressure-dependent proportionality of gas absorption. Conversely, gas diffusivity was constant and independent of exposed pressure. Gas uptake and diffusivity were the same for all samples, regardless of their thickness. The time required to attain equilibrium during gas uptake was proportional to the squares of the sample thickness, indicating that the sample behavior followed the Fickian diffusion equations.

Moreover, the logarithmic diffusivities of all PE samples decreased with increasing kinetic diameter in the order of *D*_He_ > *D*_H2_ > (*D*_O2_ ≒ *D*_Ar_) > *D*_N2_. In all tested substances, logarithmic solubility linearly increased with increasing critical temperature as follows: *S*_Ar_ > *S*_O2_ > *S*_N2_ > *S*_H2_ > *S*_He_.

After calculating the permeability of PE using the solution–diffusion mechanism, the correlation between the volume fraction of the amorphous phase and the fractional free volume was investigated. Both the volume fraction of the amorphous phase and fractional free volume ($\varphi_{a}$ and FFV) were related to permeability. From this relationship, the reduction in the amorphous phase in PE was found to be accompanied by a reduction in the free volume; this was a novel result.

In this study, PE was selected as an appropriate candidate for gas permeability characterization because its simple molecular structure allowed the correlations of gas permeation properties by considering only crystallinity. On the other hand, because other polymers have higher-order structures such as complex chemical structures, copolymers and blends, the permeation properties cannot be solely characterized through the effect of crystallinity. Thus, to understand the permeation properties for polymers of various types, future studies should be conducted in view of the microstructures, including crystallinity.

## Figures and Tables

**Table 1:** Density and degree of crystallinity measured for the three PE samples with different thicknesses.

PE Material	Thickness (mm)	Density (g/cm^3^)	Crystallinity (%)
LDPE	1.64 ± 0.03	0.91 ± 0.04	30 ± 3
	3.24 ± 0.06		
	4.93 ± 0.10		
UHMWPE	1.64 ± 0.08	0.94 ± 0.06	42 ± 3
	3.24 ± 0.16		
	4.93 ± 0.25		
HDPE	1.64 ± 0.03	0.97 ± 0.04	62 ± 6
	3.24 ± 0.06		
	4.93 ± 0.10		

**Table 2:** Diffusivity and solubility for the five gases in LDPE, UHMWPE and HDPE.

Sample	Diffusivity [×10^−11^ m^2^/s]					Solubility [mol/m^3^∙MPa]				
	H_2_	He	N_2_	O_2_	Ar	H_2_	He	N_2_	O_2_	Ar
LDPE	33.4	58.4	2.59	3.87	3.42	6.77	2.50	8.32	16.0	19.9
UHMWPE	23.3	55.9	1.78	2.26	2.36	7.04	2.30	8.22	14.4	19.7
HDPE	22.5	45.7	1.53	2.02	1.99	4.43	1.62	5.30	10.1	12.4

**Table 3:** Kinetic diameters and critical temperatures for gas molecules.

Gas	H_2_	He	N_2_	O_2_	Ar
Kinetic diameter [nm]	0.289	0.260	0.364	0.346	0.343
Critical temperature [K]	32.98	3.35	126.19	154.58	150.70

**Table 4:** Permeation parameters determined from the volume fraction of the amorphous phase and fractional free volume.

	Volume Fraction of Amorphous Phase			Fractional Free Volume	
Gas(j)	*D_a,j_* [×10^−11^ m^2^/s]	*S_a,j_* [mol/m^3^∙MPa]	*P_a,j_* [×10^−10^ mol/m∙s∙MPa]	*P_FFV,j_* [×10^−10^ mol/m∙s∙MPa]	*B_j_*
H_2_	43.4 ± 3.8	10.1 ± 0.7	42.2 ± 2.3	7573 ± 1599	1.94 ± 0.07
He	86.8 ± 6.8	3.54 ± 0.11	29.5 ± 2.8	4229 ± 2870	1.86 ± 0.24
N_2_	3.27 ± 0.23	12.1 ± 0.23	3.88 ± 0.13	826.0 ± 142.9	2.00 ± 0.06
O_2_	4.57 ± 0.53	22.4 ± 0.5	10.3 ± 1.0	2859 ± 1610	2.09 ± 0.21
A_r_	4.31 ± 0.30	28.7 ± 1.4	12.2 ± 0.3	2611 ± 633	2.01 ± 0.09

## Data Availability

Not applicable.
